# Enhancing esophageal cancer detection using a deep learning framework and a novel spectrum-aided vision enhancer for virtual narrow band imaging

**DOI:** 10.1038/s41598-026-52501-y

**Published:** 2026-05-20

**Authors:** Yu-You Tsai, Kun-Hua Lee, Arvind Mukundan, Riya Karmakar, Yaswanth Nagisetti, Danat Gutema Seyoum, Seint Lei Naing, Chien-Wei Huang, Hsiang-Chen Wang

**Affiliations:** 1 Department of Gastroenterology, Kaohsiung Armed Forces General Hospital, 2, Zhongzheng 1st.Rd., Lingya District, Kaohsiung City, 80284 Taiwan; 2 Department of Medicine, National Defense Medical University, No.161, Sec. 6, Minquan E. Rd., Neihu District, Taipei City, 11490 Taiwan; 3 Department of Mechanical Engineering, National Chung Cheng University, 168, University Rd., Min Hsiung, 62102 Chia Yi Taiwan; 4 Department of Trauma, Changhua Christian Hospital, Changhua, No. 135, Nanxiao St., Changhua City, 50006 Changhua County Taiwan; 5 Department of Biomedical Imaging, Chennai Institute of Technology, Sarathy Nagar, Chennai, 600069 Tamil Nadu India; 6 Department of Computer Science Engineering, School of Engineering and Technology, Sanjivani University, 423603 Singnapur, India; 7 Department of Integrated Bachelor of Technology, School of Engineering and Technology, Sanjivani University, 423603 Singnapur, India; 8 Department of Computer Science, Vel Tech Rangarajan Dr. Sagunthala R&D Institute of Science and Technology, No.42, Avadi-Vel Tech Road Vel Nagar, Avadi, Chennai, 600062 Tamil Nadu India; 9 School of Information, Computer, and Communication Technology, Sirindhorn International Institute of Technology, Thammasat University, 131 Moo 5, Tiwanon Rd, Bangkadi, Meung, Bangkok, 12000 Patumthani Thailand; 10 Department of Nursing, Tajen University, 20, Weixin Rd., Yanpu Township, 90741 Pingtung County Taiwan; 11 Department of Medical Research, Dalin Tzu Chi Hospital, Buddhist Tzu Chi Medical Foundation, No. 2, Minsheng Road, Dalin, Chiayi, 62247 Taiwan; 12 Development of Technology, Hitspectra Intelligent Technology Co., Ltd., Kaohsiung, 80661 Taiwan

**Keywords:** Deep learning, Medical image processing, Esophageal cancer, Spectrum-aided vision enhancer (SAVE), Virtual NBI, Computer-aided diagnosis (CAD), Object detection, Cancer, Computational biology and bioinformatics, Engineering, Mathematics and computing

## Abstract

Esophageal cancer is a highly aggressive malignancy where early detection is critical for survival. However, early-stage lesions typically present subtle mucosal changes that are difficult to identify using standard White Light Imaging (WLI), and hardware-based Narrow Band Imaging (NBI) is not universally available. In this study, we propose a novel image processing algorithm termed the Spectrum-Aided Vision Enhancer (SAVE) to address these challenges in computer-aided diagnosis (CAD). Leveraging hyperspectral data principles, SAVE transforms standard WLI endoscopic images into enhanced, NBI-like representations, significantly improving mucosal contrast and lesion visibility without requiring additional hardware. To validate the efficacy of this approach for medical image analysis, we utilized a dataset of Squamous Cell Carcinoma (SCC) and dysplasia. We conducted a comprehensive comparative analysis using five state-of-the-art deep learning models: YOLOv8, InceptionV3, Inception-ResNet-V2, ConvNeXt-V2, and MobileNetV2. Experimental results demonstrate that models trained on SAVE-enhanced images significantly outperform those trained on traditional WLI in both classification and detection tasks. This study presents a cost-effective, software-driven solution that integrates advanced image processing with deep learning, offering a robust tool for the automated screening of esophageal malignancies.

**Supplementary Information:**

The online version contains supplementary material available at 10.1038/s41598-026-52501-y.

## Introduction

Gastroenterology is a branch of medicine that studies the digestive system, its disorders, treatment, diagnosis, and management of gastrointestinal bleeding. Gastrointestinal (GI) tract cancer encompasses a variety of cancers which can cause GI bleeding and are life-threatening. Some of them are stomach cancer, esophageal cancer, colon cancer, small intestine cancer, and others^1^. According to recent statistics, esophageal cancer is the seventh most common cancer, with 572,000 new cases annually, and it ranks 6th in mortality with more than 509,000 deaths. The highest regional reports are indicated in Eastern Asia, with Mongolia and China mentioned among the top five worldwide^2^. Squamous cell carcinoma (SCC) and esophageal adenocarcinoma (EAC) are the two main histological subtypes of this male-dominant aggressive malignancy, each indicates different geographical and racial distribution patterns^3^. SCC is the most common subtype, which is mostly reported in the region of Asia and Africa, whereas EAC is more frequently observed in northern America and Europe and is associated with obesity and gastroesophageal reflux disease (GERD)^4^. Epidemiological research has shown that smoking and drinking alcohol are a significant risk factor for SCC.

The two main abnormalities of esophageal cancer, which are dysplasia, and SCC have their own character and identification. SCC mostly affects the middle third of the esophagus and less frequently it can also affect the lower part. However, the cases are rare in the upper esophagus. Squamous dysplasia is a histological lesion kept within the epithelium, characterized by both architectural and cytologic abnormal growth. Architectural changes are described as the loss of cell polarity and lack of surface maturation. Cytologic abnormalities include enlarged nuclei, hyperchromasia (black stained nuclei), pleomorphism (variation in cell shape and size), and increased or abnormal mitotic activity^5^. Metaplasia is a process in which the normal squamous cell layer of the esophagus is changed to columnar cells.

Over the past decade, artificial intelligence (AI) applications especially machine learning (ML) have achieved excellent advancement in the field of computer vision^6^. The study focused on classifying the early stages of esophageal cancer is utilizing ResNet50 and a support vector machine (SVM) to classify Barrett’s esophagus (BE) and esophagitis^7^. In the other study done in 2021, the Inception-v2 model was used to classify GI computed tomography (CT) images to diagnose esophageal cancer^8^. To detect and classify various types of esophageal tissue abnormalities white light imaging has been a very common endoscopic technique used for medical image analysis and showed less accurate results than computer-aided detections CADe^9^. In the comparison study of the diagnostic capacity of convolutional neural networks (CNN) and endoscopists, the result demonstrates that CNN is showing better results in detecting early cancer. CNN was organized by utilizing 13,584 endoscopic images from 2639 lesions of cancer and the result is curated using a test dataset of 2940 images from 140 classes^10^. In the performance comparison of AI versus expert endoscopy, images of 23,746 from 1544 cases of esophageal SCCs and images of 4587 from 458 noncancerous tissues and normal tissues were utilized to build an AI system. Since, the accuracies, specificities, and sensitivities for the identification of SCC were high in percentage, the AI system performed well^11^. The major limitations of WLI shown in these comparisons include being less sensitive to point out the mucosal and vascular changes that are indicative of early or serious stages of cancer, using broad-spectrum light which doesn’t use specific light wavelength to identify the lesions clearly, and having a longer learning curve^12^. However, most of the studies use WLI images which utilize three primary color bands, integrating these highly performed AI systems with hyperspectral imaging (HSI) is an innovative way of capturing images beyond WLI and brings high assurance in medical fields. For instance, the five-layered CNN with the dataset of 300 HSI was trained and fine-tuned then it achieved 94.3% accuracy^13,14^.

HSI which captures and processes information across the electromagnetic spectrum is an emerging technique for medical imaging^15^. Basically, HSI operates by capturing images based on wavelength variation using spectroscopic techniques. The photons scattering in the blood vessel is recorded to produce spectral images across narrow spectral bands, particularly resulting in multiple bands. For specific bands, the relative amount of light absorbance or reflectance which helps to increase the contrast of cancerous tissues for each image will be done. This information is compiled into a finite 3D volume with two spatial dimensions (X rows and Y columns) and one spectral dimension (λ wavelength), known as a hyperspectral image, hyperspectral (HS) cube, or hypercube^16,17^. The working principle of an HSI that makes use of particular cameras for implementation purposes is divided into main 3 techniques: spectral scanning, spatial scanning, and snapshot method^18^. However, these three techniques are expensive and not easy to use because they require highly specialized cameras such as pushbroom which are more complex than normal imaging systems. They also need high-quality optics for the spectrometer or filters to separate and capture specific wavelengths, and they use precise calibration to ensure accurate data. Hence, the more specific technique called narrow-band imaging (NBI) which is obtained from HSI conversion, helps in getting more convenient and high-resolution images.

NBI is an endoscopic method that makes use of green and blue lights to highlight mucosal and submucosal vasculature^19^. The shorter wavelength of these visible lights (415 nm and 540 nm ) has low tissue permeability which makes it a highly preferable way for identifying mucosal surface structure^20,21^. Because of the strong ability to be absorbed by hemoglobin and its low penetration behavior blue light is used effectively in superficial layers of mucosa. The green light has a slightly longer wavelength and can penetrate a bit deeper than the blue light utilized to visualize the submucosal blood vessel^22^. This NBI method was described in 2003 for the first time and helps to facilitate the detection of early SCC^23^. The study which was done in 1780 esophageal cancer images that include 935 NBI images and 845 WLI, NBI showed better results for the SCC group and good estimation for the normal group in comparison^24^. Similar research was done at the Kaohsiung Medical University for 45 patients and proved that the diagnosis of NBI has greater sensitivity compared with that of white light endoscopy^25^.

Therefore, in this work, NBI has been combined with HSI and used to select a few spectrums to enhance the contrast of esophageal cancer identification, particularly dysplasia before it turns into SCC. The CAD system is the best way to increase the visualization of esophageal tissue and enable accurate differentiation among normal, dysplasia, and SCC especially if it integrates with SAVE. By applying the SAVE model and training different ML models for the given dataset, this work focuses on creating a robust model that can identify and prevent pre-cancerous conditions. For the given dataset the captured WLI images are converted into SAVE and made available for further training.

## Methods

### Dataset

The dataset used in this study comprises a total of 5370 images, which were obtained from 185 unique patients (50 patients with dysplasia, 65 with SCC, and 70 with normal esophageal tissue). To ensure rigorous evaluation and prevent data leakage, dataset partitioning was strictly conducted at the patient level prior to the generation of SAVE images. All frames associated with a single patient were assigned exclusively to either the training, validation, or testing set using a 70:20:10 ratio. This protocol ensures that the model performance is validated on images from patients that the network has never encountered during training, thereby providing a more accurate assessment of its clinical diagnostic potential. Each split of the dataset contains three main classes and specifically the training dataset contains 3 classes with image distribution of 1420, 1175, and 1160 for the dysplasia, normal, and SCC respectively for both WLI and SAVE images (overall distribution of images per class is summarized in Table S2). All methods were carried out in accordance with relevant guidelines and regulations. The experimental protocols involving human subjects were approved by the Institutional Review Board of Kaohsiung Armed Forces General Hospital (Approval No. KAFGHIRB 114 − 022). Due to the retrospective nature of the study and the use of de-identified images, the requirement for informed consent was waived by the Institutional Review Board of Kaohsiung Armed Forces General Hospital.

### SAVE

This study introduces the Spectrum-Aided Vision Enhancer (SAVE), a novel image processing technique designed to transform standard White Light Imaging (WLI) into hyperspectral-enhanced representations (HSI and SAVE images). The primary objective of SAVE is to augment the diagnostic capability of conventional endoscopy by recovering detailed spectral information that is typically lost in traditional RGB imaging. The core of the SAVE mechanism lies in the precise calibration of endoscopic RGB data to match the reflectance spectra obtained from a spectrometer. To establish a robust mapping between the WLI images and the ground-truth spectral data, we utilized a Macbeth Color Checker (X-Rite Classic), which contains 24 standardized color patches representing natural reflectance spectra. The calibration pipeline begins by converting the raw endoscopic images from the standard sRGB color space into the CIE 1931 XYZ color space, a device-independent standard in colorimetry where X, Y, and Z correspond to the trichromatic response functions. To ensure high-fidelity color reproduction, we applied a series of correction algorithms to address common imaging artifacts, including non-linear sensor response, dark current noise, imperfect color separation, and lens distortion. The corrected XYZ values (XYZ_correct_) are derived through this rigorous calibration process. The transformation from raw RGB to the initial XYZ space is formulated in Eq. (1), while the subsequent error correction and calculation of the final XYZ_correct_ are defined in Eq. (2).

1 $$\:\left[C\right]=\left[XY{Z}_{Spectrum}\right]\times\:pinv\left(\left[V\right]\right)$$

2 $$\:\left[XY{Z}_{Correct}\right]=\left[C\right]\times\:\left[V\right]$$

Following the initial color space conversion, the calibration process necessitates a rigorous alignment between the generated SAVE representations and the clinical gold standard—Narrow Band Imaging (NBI) captured by Olympus endoscopes. To achieve this, we employed Principal Component Analysis (PCA) to extract the most significant features from the high-dimensional reflectance spectra. This step is crucial for reducing data dimensionality while preserving essential spectral information, thereby optimizing computational efficiency. Our PCA analysis revealed that the first six principal components were sufficient to capture 99.64% of the total variance in the spectral data. This finding confirms that a low-dimensional representation can effectively model the complex reflectance properties of esophageal tissue. To validate the accuracy of our color calibration, we calculated the Root Mean Square Error (RMSE) between the corrected image values (XYZ_correct_) and the ground-truth spectrometer measurements (XYZ_spectrum_). The resulting mean RMSE of 0.19 indicates a minimal color difference, demonstrating the high fidelity of our calibration pipeline.

The successful calibration serves as the foundation for converting standard WLI images into Hyperspectral Imaging (HSI) formats and subsequently into virtual NBI (SAVE) images. To formalize this conversion, we conducted a multiple regression analysis to model the relationship between the input WLI data and the target spectral components. This analysis yielded a transformation matrix, defined in Eq. (3), which serves as the mathematical kernel for mapping the broad-spectrum WLI signals to the specific narrow-band wavelengths required for SAVE. By applying this matrix, we can synthetically reconstruct tissue visualization that mimics the contrast-enhancement properties of hardware-based NBI, effectively highlighting vascular structures and mucosal patterns.

3 $$\:\left[M\right]=\left[Score\right]\times\:pinv\left(\left[{V}_{Color}\right]\right)$$

The analog spectrum derived from the corrected XYZ values Eq. 4 demonstrated minimal color discrepancies compared to real measurements, supporting the system’s accuracy:

4 $$\:{\left[SSpectrum\right]}_{380\sim780nm}=\left[EV\right]\left[M\right]\left[{V}_{Color}\right]$$

Upon reconstructing the hyperspectral data from RGB inputs, the subsequent phase involved synthesizing virtual NBI representations and validating their fidelity against clinical standards. We utilized the Olympus NBI system as the ground truth benchmark, comparing its output directly with our algorithmically generated SAVE images. This comparison was facilitated using the 24-patch color checker to ensure consistent colorimetric evaluation. To quantify the perceptual similarity between the synthetic SAVE images and the hardware-captured NBI images, we computed the CIEDE2000 color difference metric (ΔE_00_) for each color patch. The initial analysis yielded a mean ΔE_00_ of 2.79, indicating a high degree of visual resemblance with only minor deviations. These discrepancies were primarily attributed to inherent differences in the illumination spectra, reflectance properties, and color-matching functions between the simulated environment and the physical endoscopic hardware. A critical observation was that the spectral mismatch was most pronounced in the 450–540 nm wavelength range, which is vital for visualizing superficial mucosal capillaries. To mitigate this and further minimize the color divergence, we implemented a secondary calibration layer utilizing the Cauchy-Lorentz distribution. This statistical model, defined in Eq. (5), is particularly effective for characterizing spectral resonance and was applied to fine-tune the system’s spectral response. By adjusting the distribution parameters, we significantly reduced the spectral deviation, ensuring that the SAVE images provided a clinically accurate emulation of the NBI modality.

5 $$\:f\left(x;{x}_{0},\gamma\:\right)=\frac{1}{\pi\:\gamma\:\left[1+{\frac{\left(x-{x}_{0}\right)}{\gamma\:}}^{2}\right]}=\frac{1}{\pi\:}\left[\frac{\gamma\:}{\left(x-{x}_{0}\right)2+{\gamma\:}^{2}}\right]$$

**Fig. 1:**
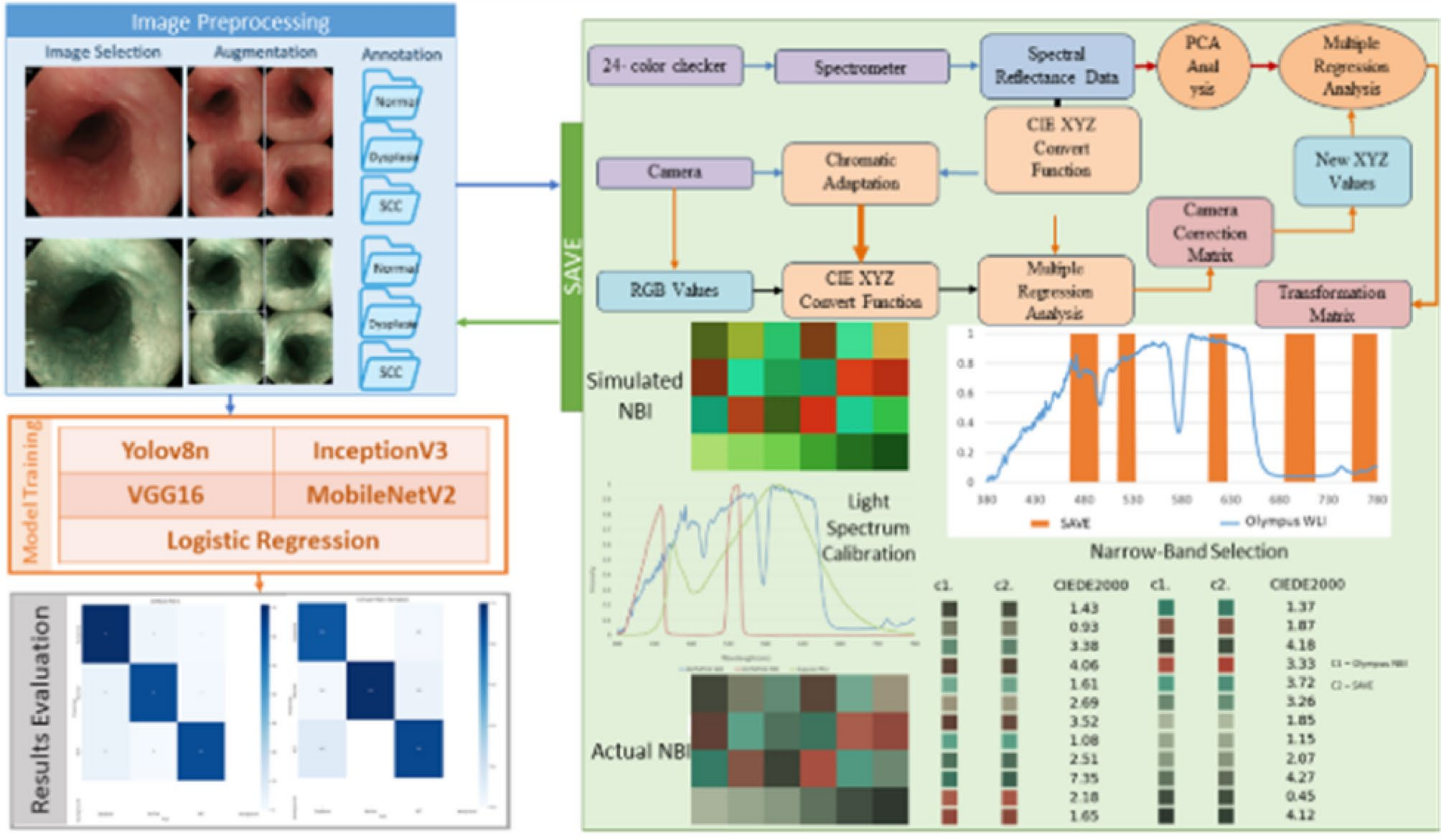
The process of converting WLI into SAVE.

To refine the light spectrum parameters, we employed the dual annealing optimization algorithm. This stochastic approach integrates the robustness of Classical Simulated Annealing (CSA) with the efficiency of Fast Simulated Annealing (FSA), allowing for a global search of the optimal spectral configuration. Following this optimization, the mean CIEDE2000 color difference was stabilized at 5.36. While this value is slightly higher than the initial calibration, it accounts for the complex post-processing inherent in the Olympus system—specifically, the introduction of brownish tones alongside the primary hemoglobin absorption peaks (415 nm and 540 nm) to enhance tissue realism. To rigorously assess the fidelity of the SAVE-generated images against the gold-standard NBI, we utilized a triad of image quality metrics: Structural Similarity Index Measure (SSIM), Entropy, and Peak Signal-to-Noise Ratio (PSNR). The SSIM analysis yielded a similarity score of 94.27%. To rigorously assess the fidelity, the SAVE-generated images were compared against the Olympus hardware NBI images as the ground truth. During the calibration phase, this comparison was performed using the 24 standardized color patches to ensure pixel-to-pixel correspondence and minimize errors from external lighting or motion artifacts. The metrics (SSIM, PSNR, and Entropy) were calculated over the entire spatial dimensions of the color patches and representative mucosal frames to validate that the SAVE mechanism maintains global structural integrity (94.27% similarity) and informational richness. This high correlation confirms that the SAVE mechanism successfully preserves the structural details and textural information of the mucosal surface, making it a reliable surrogate for hardware NBI. Entropy was calculated to evaluate the richness of image information. The results showed a marginal difference of only 0.37% between the SAVE and Olympus NBI images, indicating that our method maintains the textural complexity required for diagnosis. The PSNR, a standard metric for reconstruction quality, reached 27.88 dB. This value suggests that the SAVE images retain high signal fidelity with minimal noise introduction during the transformation process. The comprehensive workflow, from initial RGB input to the final optimized SAVE output, is schematically illustrated in Fig. 1.

**Fig. 2:**
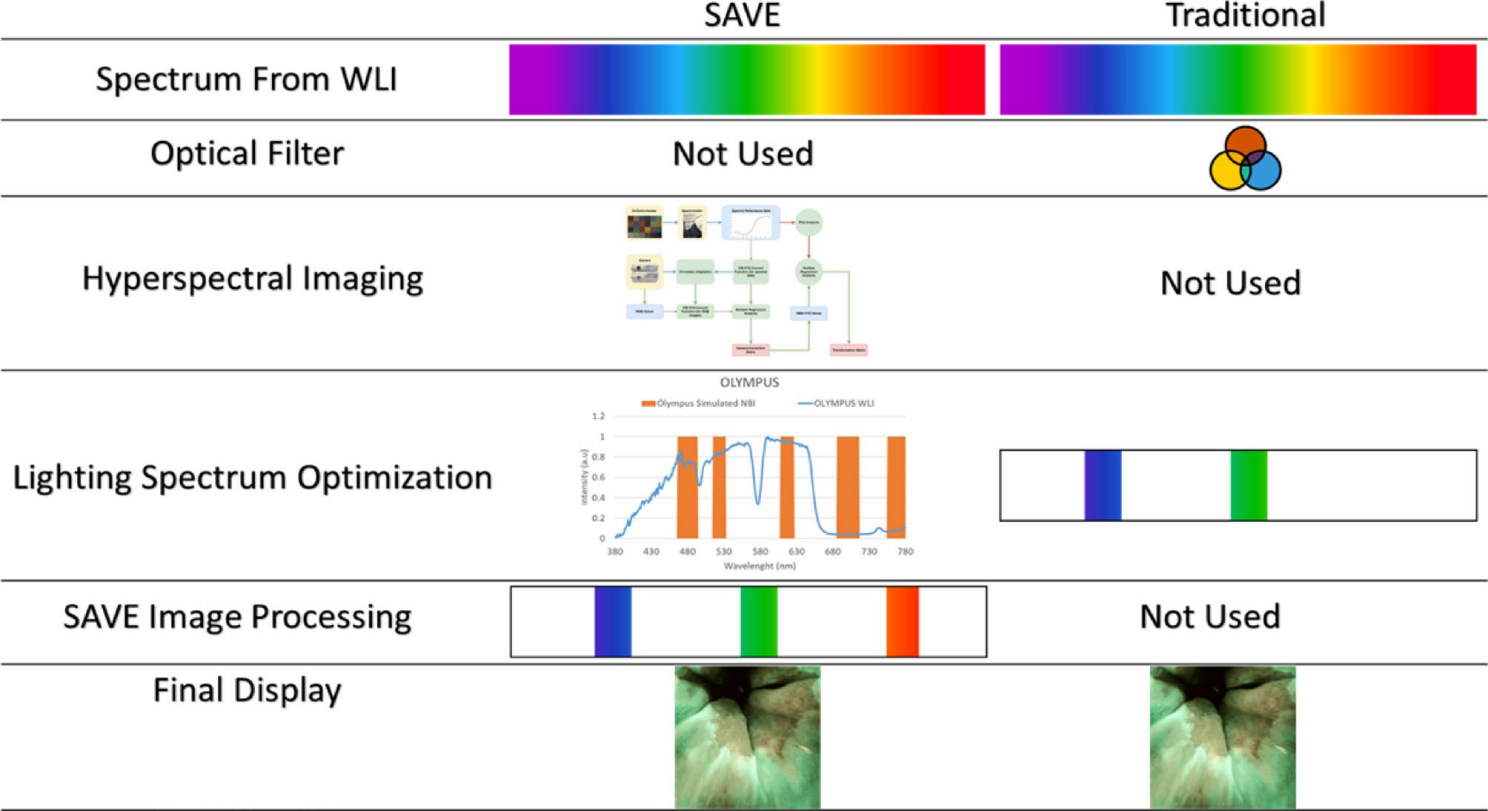
Novelty of the proposed SAVE algorithm compared with the traditional methods.

A schematic comparison illustrating the originality of the proposed SAVE framework relative to existing hyperspectral and virtual narrow-band imaging approaches is presented in Fig. 2. Traditional HSI methods often employ specialized spectral cameras to directly measure a limited number of narrow wavelength bands on tissue, necessitating specialized hardware and complex acquisition procedures. In contrast, the proposed SAVE framework reconstructs hyperspectral-inspired spectral data from standard RGB white-light endoscopic pictures utilizing a calibrated colorimetric workflow. SAVE approximates color spectrums without specialized sensors by interpolating RGB values into the CIE XYZ color space and correlating the outcomes with observed spectral reflectance through Macbeth Color Checker calibration. The reconstructed spectral representation is further refined using principal component analysis and spectral optimization based on the Cauchy-Lorentz distribution to imitate the spectral behaviour of Olympus narrow-band imaging. This method allows SAVE to generate virtual NBI-like pictures from conventional WLI images, ensuring compatibility with current clinical endoscopic systems and facilitating integration with deep learning-based computer-aided diagnosis models.

### DL algorithm

#### YOLOv8

The first YOLO model was introduced in 2015 by Joseph Redmon in a C-based repository named Darknet during his Ph.D. studies at the University of Washington. Since then, the community has continued developing and enhancing the subsequent YOLO version^26,27^. Ultralytics the community that introduced YOLOv5 released YOLOv8 in January 2023. This new version supports different computer vision tasks including object detection, object segmentation, tracking, and classification. By targeting the specific need of the task YOLOv8 developed with five different variants including YOLOv8n (nano), YOLOv8s (small), YOLOv8m (medium), YOLOv8l (large), and YOLOv8x (extra-large)^28,29^.

The backbone responsible for the feature extraction of the input images is one of the main components of the YOLOv8 architecture. By making use of the path aggregation network (PANet) with feature pyramid network (FPN) the other architecture called neck detects objects at various levels(an illustration of the YOLOv8 architecture used in this study is shown in Fig. S1). The detection head of YOLOv8 is where the final prediction is done and for tasks like object classification and regression tasks decoupled head is utilized^30,31^. For different tasks, YOLOv8 uses different Loss functions. The classification loss utilizes Variational Focal Loss (VFL), and the regression employs a combination of Distribution Focal Loss (DFL) and Complete IoU (CIoU) Loss. Unlike Focal Loss (FL) and Quality Focal Loss (QFL), which are symmetric, VFL uses an asymmetric weighting technique to find out the solution of the imbalance between negative and positive samples.

6 $$\:VF{L}_{\left(p,q\right)}=\left\{-q\left(q\:\mathrm{log}\mathrm{log}\left(P\right)+\left(1-q\right)\mathrm{log}\left(1-p\right)\right),q>0-\alpha\:{P}^{y}\mathrm{log}\left(1-p\right),q=0\right\}$$

Where VFL stands for variational focal loss, p represents the label and q is a value calculated by normalized alignment matric if a positive sample is taken.

#### InceptionV3

Transfer learning is a way of solving a new problem based on the previous learning history of the model. The main characteristic of the neural network is that the layer that is closer to the output is updated based on the new data, while the remaining hidden layers are kept unchanged^32^. InceptionV3 is one of the transfer learning models built as an advanced version of Google Net (InceptionV1) which achieved the first prize in the 2014 ILSVRC Competition^33^. This model is the other version of Inception-V1 and Inception-V2 architectures and it’s deep CNN which is trained on low-configuration computers^34^. Moreover, the model is trained on the large-scale ImageNet dataset and has the ability to classify images into 1000 different groups^35^. The architecture of inceptionV3 can be divided into smaller convolutional kernels which has a huge effect of lowering the model’s parameter and minimizing the probability of overfitting^36^. The main architecture includes convolution, MaxPool, Average Pool, fully connected, dropout, and SoftMax^37^. The loss function of the inceptionV3 for the classification task is represented in Eq. 7.

7 $$\:L=-\:{\sum\:}_{i=1}^{c}{y}_{i}\mathrm{log}\left({x}_{i}\right)$$

Where y_i_ is the true value of class i, x^i^ is the predicted probability for class I, and C is the overall number of classes.

#### ConvNeXt-V2

ConvNeXt-V2 is a modern convolutional neural network model that aims at closing the performance gap between traditional CNN models and models based on transformers. It uses the concepts of the self-attention mechanism of transformers by a Fully Convolutional Masked Autoencoder (FCMAE) framework, which allows the model to learn more expressive and strong image features. Under this framework, portions of the input image are masked, and the network learns to replicate the missing portions, which enhances its feature-learning capacity. To facilitate this process in ConvNeXt-V2, regular convolutions in the masked regions are substituted with sparse convolutions, and the reconstruction loss of the pre-training is based on Mean Squared Error (MSE). Another major advancement in ConvNeXt-V2 is the integration of Global Response Normalization (GRN), a mechanism designed to mitigate feature collapse—a phenomenon where deep models saturate or deactivate feature maps, limiting their representational capacity. GRN enhances channel-wise feature competition by normalizing the global activation strength, thereby stabilizing the learning process. Mathematically, for a given input feature map with defined height, width, and channel dimensions, the global response is first computed by aggregating the spatial information across each channel using the L2-norm, as defined in Eq. (8)^38^:

8 $$\:G\left(X\right)=\left\{\left|\left|{X}_{1}\right|\right|,\:\left|\left|{X}_{2}\right|\right|,\dots\:,\left|\left|{X}_{c}\right|\right|,\right\}$$

Subsequently, a channel-wise normalization step is applied to compute the relative importance of each channel, denoted as N(X), as shown in Eq. (9):

9 $$\:N\left(\left|\left|{X}_{i}\right|\right|\right)=\frac{\left|\left|{X}_{i}\right|\right|}{\sum\:_{j=1}^{C}\left|\left|{X}_{j}\right|\right|}$$

Finally, the calibrated feature map is obtained by scaling the original input with learnable scaling and shifting parameters, ensuring that the network retains the flexibility to adaptively emphasize informative features. This transformation is expressed in Eq. (10):

10 $$\:{X}_{i}^{{\prime\:}}=\gamma\:\bullet\:{X}_{i}\bullet\:N\left(G\left({X}_{i}\right)\right)+\beta\:+{X}_{i}$$

Where $\:\gamma\:$ and $\:\beta\:$ are learnable parameters.

ConvNeXt-V2 was employed in this work to classify images of the esophagus (Normal, Dysplasia, and SCC) with the help of WLI and SAVE-generated hyperspectral band-selected data. Resizing was done using all the images to the size of 224 × 224 pixels prior to training. Due to the design of ConvNeXt-V2, focusing on high-accuracy classification with high generalization, the algorithm is compatible with medical images where the slightest changes in tissue texture and color can need to be detected. In order to guarantee the dependability of the assessment, 5-fold cross-validation was used, and the average performance scores across all folds were obtained. This enabled the model to be tested equally on WLI and the hyperspectral inputs.

#### Inception-ResNet-V2

Inception-ResNet-V2 is a deep convolutional neural network, which combines the multi-scale feature extracting capability of Inception modules with the stability of residual learning. The model starts with a stem block which is capable of basic feature extraction and then there are three major blocks, Inception-ResNet-A, Inception-ResNet-B, and Inception-ResNet-C. Many convolutional branches operating in parallel are contained in each block, and this aids the network to acquire features of images at various scales.

A defining characteristic of this architecture is the incorporation of shortcut connections (skip connections) within each Inception block. These connections compel the network to learn a residual mapping rather than a direct transformation, which significantly enhances training stability and mitigates the vanishing gradient problem in deep networks. The residual learning mechanism is formally defined in Eq. (11)^39^:

11 $$\:H\left(x\right)=F\left(x\right)+x$$

Where represents the input to the block, whereas () is the multi-branch convolutional output. All the blocks in the architecture are done with batch normalization and reduction in order to accelerate training and regulate model complexity. The Inception-ResNet-V2 has a number of hundreds of layers, which enables it to make both fine and complex contributions to medical images. The model was applied in this research to categorize esophageal images of the WLI and SAVE. All the images were downscaled to 224 × 224 pixels and then submitted to the network. To make the evaluation reliable, a 5-fold cross-validation procedure was performed, i.e. the dataset was split into five equal parts and model was trained and validated five times. This increased the performance comparison between the WLI and the hyperspectral images in terms of being more accurate and unbiased.

#### MobileNetV2

MobileNetV2 is one of the neural networks which is developed by Google and it runs on any device efficiently with less computational power and gives high accuracy^40^. The architecture of the model contains an inverted residual block, linear bottleneck layer, depth-wise separable convolution, ReLU6 Activation, expansion layer (1 × 1 convolution), stridden convolutions for down-sampling, global average pooling (GAP), and final fully connected layer^41,42^. The model depends on depth-wise separable convolutions which separate standard convolution into two steps: depth-wise (pre-channel filtering) and pointwise (combining information across channels). This is important to reduce the computational complexity compared to traditional convolutions while maintaining accuracy^43^. The other architecture of mobilenetv2 which are inverted residual and linear bottleneck structures significantly reduce the computational cost of convolutions making it both efficient and effective to use^44^.

## Results

**Table 1:** Performance of various ML models for a given dataset (Mean values from 5-fold cross-validation).

Models	Imaging modalities	Classes	Precision	Recall	F1 score	Specificity
YOLOv8n	WLI	Normal	78.42	87.15	82.56	85.33
		Dysplasia	93.10	85.42	89.10	97.21
		SCC	87.25	82.11	84.60	97.05
	SAVE	Normal	78.95	87.68	82.14	85.91
		Dysplasia	90.12	88.34	89.22	96.45
		SCC	86.44	76.50	81.16	94.20
InceptionV3	WLI	Normal	84.21	93.18	88.47	89.12
		Dysplasia	91.15	88.34	90.12	96.45
		SCC	97.20	88.11	92.43	98.32
	SAVE	Normal	89.34	95.12	92.14	92.45
		Dysplasia	92.11	97.23	94.60	96.21
		SCC	100	85.42	92.13	100
Inception-ResNet-V2	WLI	Normal	90.24	93.15	91.67	94.21
		Dysplasia	89.12	94.21	91.60	94.32
		SCC	94.35	85.12	89.50	97.11
	SAVE	Normal	97.12	90.45	93.67	98.24
		Dysplasia	86.42	94.35	90.21	93.11
		SCC	91.23	91.15	91.19	96.42
ConvNeXt-V2	WLI	Normal	78.12	88.23	83.14	85.45
		Dysplasia	96.45	74.12	83.91	98.23
		SCC	89.12	97.34	93.21	94.10
	SAVE	Normal	93.24	86.11	90.45	96.12
		Dysplasia	89.23	99.12	94.32	94.12
		SCC	92.14	91.32	92.15	96.45
MobileNetV2	WLI	Normal	85.12	95.23	89.89	89.23
		Dysplasia	88.34	88.12	88.23	88.11
		SCC	97.15	82.34	89.14	89.42
	SAVE	Normal	83.42	98.12	90.17	88.45
		Dysplasia	91.23	88.45	89.82	96.12
		SCC	100	82.11	90.18	100

The cross-validation that was performed in five-fold in all five deep learning models, i.e., YOLOv8n, InceptionV3, Inception-ResNet-V2, ConvNeXt-V2, and MobileNetV2, demonstrated different trends in the diagnostic performance of the models with application to WLI and SAVE imaging modalities in Table 1. This variation in precision, recall, F1-score, and specificity between the Normal, Dysplasia as well as SCC classes can be directly explained by the variation in model architecture and the quality of visual information that each imaging modality possesses. SAVE scans always provided better clarity, contrast, and structural visibility, which contributed greatly to the change of the pattern of performance in terms of classes. Fold-wise numerical results are given in Supplementary Tables S1–S25 and all fold-wise loss plots and confusion matrices are found in Figs. S1–S90.

The first one began with YOLOv8n whose lightweight structure was relatively sensitive to the gains made by SAVE imaging. There was a minor improvement in dysplasia performance under SAVE mainly because of the increase in the level of illumination and clarity of the epithelial pattern by which early-stage morphological variations, which are weak in WLI, can be detected. This has led to more increase in recall demonstrating that a smaller number of dysplastic cases were overlooked. But SCC recall declined with SAVE as greater strengthening of intermediate dysplastic features by the modality introduced minor overlaps between high-grade dysplasia and early SCC leading to misclassification at the border between the two classes. Normal class was also similar in modalities as YOLOv8n already works well with normal tissue and the extra SAVE clarity did not dramatically change the property of the class. These results indicate the consistency of YOLOv8n and also exemplify its weaknesses in discriminating closely related pathological classes in the case of adding visual complexity through SAVE. Fold-wise numerical results are given in Supplementary Tables S11–S15 and all fold-wise loss plots and confusion matrices are found in Figs. S71–S90.

InceptionV3, on the contrary, was more advantaged by SAVE images. Its multi-scale convolutional filters are based heavily on rich spatial detail and color gradient both of which are more exhibited in SAVE. The recall of dysplasia (strong) and near-perfect recall showed an improvement of values under SAVE, which highlights the fact that the latter is more accurate in the detection of small lesions. Equally, precision in SCC was 100% with SAVE, showing that more evident morphological indicators resulted in the model being able to remove false positives completely. The sharpening of the epithelial edges, and the even distribution of light in SAVE also led to improved normal tissue recognition since the model could avoid misclassifying benign abnormalities that were a common occurrence in WLI. Collectively, these improvements affirm that the InceptionV3 architecture is extremely compatible with the sophisticated spectral and structural data delivered by SAVE which allows to perform more confident classification in all tissue types. Fold-wise numerical results are given in Supplementary Tables S1–S5 and all fold-wise loss plots and confusion matrices are in Figs. S21–S40.

Inception-ResNet-V2 also produced good and robust performance in both modalities, however, SAVE provided some additional benefits that enhanced the reliability of the classification. This architecture has residual connections together with inception modules which makes it especially effective at refining intermediate features. The highest improvement was observed with the Normal class with SAVE whereby the precision was enhanced significantly. This was the case because SAVE lowers the appearance of shadows, glare and minor inflammatory alterations which may appear like pathology and enhances the confidence of the model to recognize non-pathological mucosa. Dysplasia performance was also very strong but the precision was very slight because SAVE tended to exaggerate benign textural changes which made the model over-detect dysplasia in normal areas. It was observed that SCC performance was also balanced, with both precision and recall improving or retaining high levels due to the fact that SCC morphology is refined obviously in SAVE and with ease in the deep layers of the feature extraction. The findings highlight the versatility of the Inception-ResNet-V2 that successfully used the better visual cues of SAVE to sharpen the predictions without causing instability. Fold-wise numerical results are given in Supplementary Tables S16–S20 and all fold-wise loss plots and confusion matrices are in Figs. S61–S70.

ConvNeXt-V2 model showed the most drastic improvements with SAVE and particularly with dysplasia class. This model is based on the transformer-style architectural ideas and relies on the detailed texture patterns and long-range dependencies which are better offered by SAVE imaging than by WLI. Recall of dysplasia increased significantly when under WLI, it was low and when under SAVE, it was almost perfect, which is that ConvNeXt-V2 needs high-quality structural and textural detail in order to identify early or mild lesions. Normal and SCC classes were also benefitted and displayed more equalized values of precision and recall with SAVE. WLI weaknesses of irregular brightness, contrast, and lesser expression of gentle architectural distortion were barriers to the generalizability of this architecture in some classes. SAVE addressed these errors and enabled ConvNeXt-V2 to see the tissue patterns in a more holistic and correct way. Fold-wise numerical results are given in Supplementary Tables S21–S25 and all fold-wise loss plots and confusion matrices are in Figs. S1–S20.

Lastly, even though MobileNetV2 is lightweight, it also showed significant gains with SAVE. This model suffers a shortcoming of not being able to extract features of WLI limited features of distinguishing dysplasia and borderline pathology. To counter this, SAVE made available more explicit micro-structural characteristics that made the boundaries of decisions easier to find in the model. The Dysplasia F1-scores were better and SCC precision was 100% indicating that MobileNetV2 was able to reliably discriminate between advanced malignant appearances when assisted by high-quality imaging. There was also the improvement of the Normal class as SAVE reduced noise, asymmetric lighting, and other minor mucosal artifacts that previously led to a false positive. Therefore, MobileNetV2 has shown that also small models can significantly benefit the added structural and spectral cues of SAVE that can be used to achieve performance in some classes similar to much larger designs. Fold-wise numerical results are given in Supplementary Tables S6–S10 and all fold-wise loss plots and confusion matrices are found in Figs. S41–S60.

**Fig. 3:**
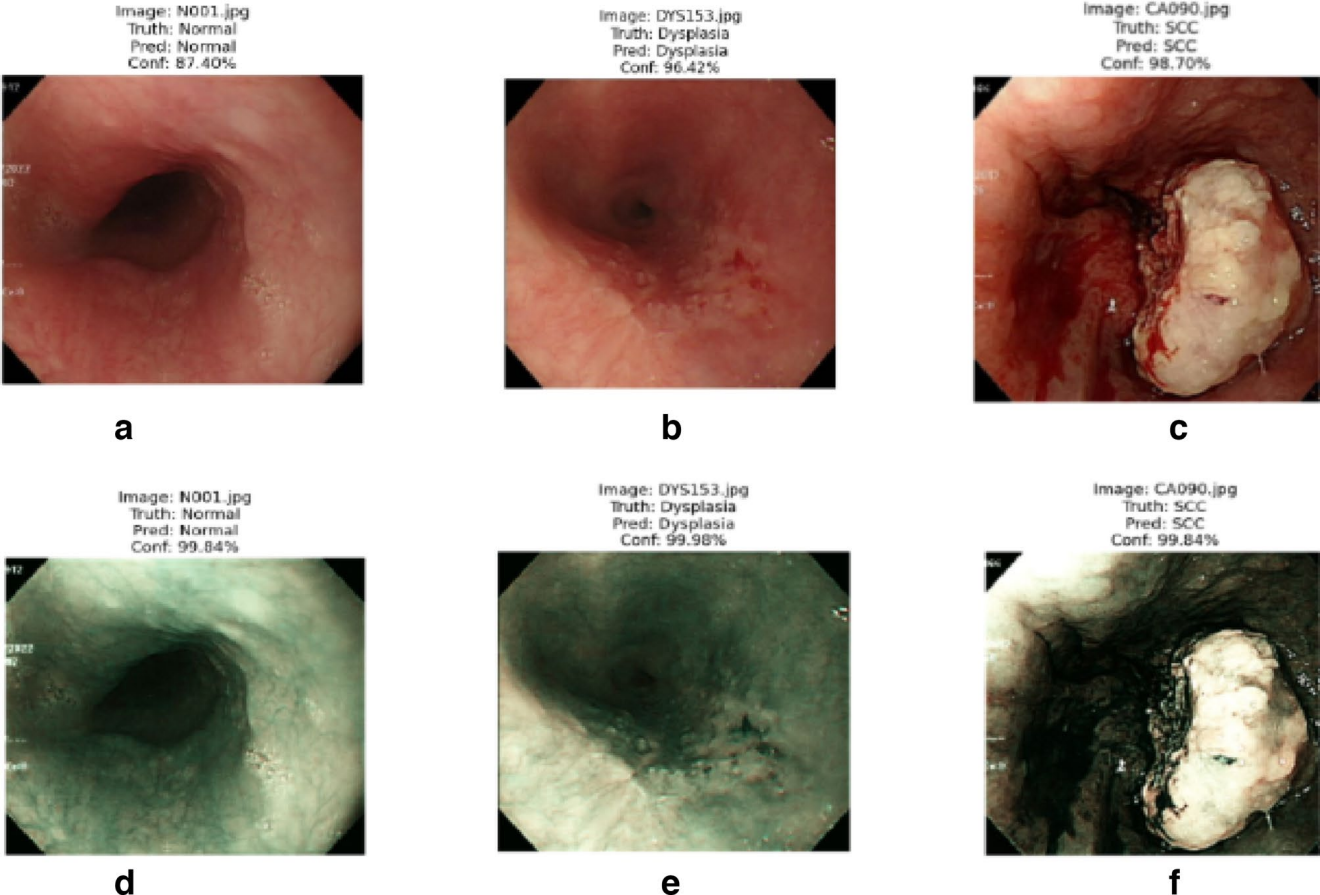
The prediction results of the WLI and SAVE model for the various classes of esophageal cancer. (**a**) The WLI image of the normal class; (**b**) The WLI image of the dysplasia class; (**c**) The WLI image of the SCC class; (**d**) The SAVE image of the normal class; (**e**) The SAVE image of the dysplasia class; (**f**) The SAVE image of the SCC class.

To enhance the statistical foundation for comparing imaging modalities, we conducted additional statistical analyses based on the F1-scores reported in the five tested deep learning architectures. The mean F1-score for models and classes was WLI 88.10 ± 3.33 and SAVE 90.10 ± 3.66, indicating an approximate 2.3% improvement in results using SAVE enhanced photos. To assess the degree of bias in performance favoring the SAVE modality, a paired Wilcoxon signed-rank test was performed on the model-level data, yielding W = 1 (p = 0.19). Although the statistical significance is low due to the limited number of evaluated topologies, the effect size (Cohen’s d = 0.57) indicates a moderate level of practical enhancement associated with the utilization of SAVE-based inputs. The evidence suggests that the additional spectral and structural data incorporated by the SAVE algorithm may improve model performance across various neural network architectures (as qualitatively illustrated in Fig. 3), and that more comprehensive datasets and statistical analyses at the prediction level will be required to perform more rigorous significance tests.

To evaluate the potential for clinical translation, we benchmarked the computational efficiency of the SAVE algorithm and the integrated CAD pipeline. The evaluation was performed on a medical-grade workstation equipped with a 12th Gen Intel Core i7-12700 F CPU, 16 GB of RAM, and an NVIDIA GeForce RTX 3070 Ti GPU (8 GB VRAM).

We measured the processing latency and throughput (FPS) across 6138 frames at a high-definition resolution of 1920 × 1080. As summarized in Table 2, the SAVE algorithm demonstrates exceptional efficiency, requiring only 8.3 ms per frame and achieving a throughput of 120.6 FPS. When coupled with the YOLOv8 detection framework, the full pipeline operates at 21.4 FPS with a total latency of 46.7 ms.

**Table 2:** Computational performance of the SAVE algorithm and the integrated CAD pipeline.

Module	Latency (per frame)	Throughput (FPS)
SAVE algorithm	8.3 ms	120.6
YOLO detection	38.4 ms	26
Full pipeline (SAVE + YOLO)	46.7 ms	21.4

## Discussion

The novelty of our methodology lies in its software-driven approach to image enhancement. Recent studies, such as the multimodal fusion networks reported in iScience^45^ and multi-task learning frameworks in Scientific Reports^46^, focus primarily on architectural innovations to handle complex gastrointestinal datasets. As summarized in Table 3, while effective, these methods often require significant computational resources or multiple imaging modalities. In contrast, the SAVE algorithm leverages hyperspectral principles to recover spectral information lost in standard RGB imaging. This provides a cost-effective alternative to hardware-based NBI without the need for specialized filters. Furthermore, while segmentation approaches like the UNet++-based study in JEST^47^ excel at delineating lesions, our framework enhances the underlying mucosal contrast, thereby providing higher-quality input for such downstream segmentation and classification tasks.

**Table 3:** Structured comparison of the SAVE + Deep Learning framework with recent state-of-the-art studies.

Study	Task type	Dataset characteristics	Key methodology	Key findings
Current study (SAVE)	Classification & detection	5370 images from 185 patients (Normal, Dysplasia, SCC)	Spectrum-Aided Vision Enhancer (SAVE): Software-driven virtual NBI	Significantly outperformed WLI in all models (YOLO, ConvNeXt, etc.).
iScience^45^	Diagnosis	Multimodal clinical data	Multimodal–multiscale hybrid fusion	High diagnostic accuracy but requires multiple data inputs.
JEST^46^	Segmentation	Early esophageal cancer images	UNet + + architecture	Effective for early lesion boundary identification.
Sci. Rep.^47^	Multi-lesion diagnosis	General gastrointestinal (GI) dataset	Deep multi-task learning frameworks	Robust generalization across various GI pathologies.

The result of this study demonstrates that the SAVE model has better results than the conventional one. Hence by enhancing the early identification of esophageal cancer, the SAVE model can contribute to better patient outcomes, as identifying and treating early cancer can prevent the spread of cancer into the severe stage. Kaohsiung Medical University Chung-Ho Memorial Hospital used as the only source of the dataset which could limit the model’s generalization. Incorporating data from different sources like multiple hospitals, different regions, races, and age groups would help to consider the geographical and genetic variability of esophageal cancer and races could increase the model’s capacity to find esophageal cancer across various demographic groups. Even though the scope of the dataset is somewhat limited to identifying and diagnosing all the types of esophageal cancer, this study aimed to establish a benchmark for ML models applied to esophageal cancer detection. Having research done on SCC which is the main type of esophageal cancer type is very crucial, as it represents both ends of the spectrum early, hard-to-detect cases (dysplasia), and severe, late-stage cases of the disease (SCC). the other important types of esophageal cancer that are not mentioned in this work include Barrett’s esophagus and high- and -low-grade dysplasia. Barrett’s esophagus is a stage in which the normal lining of the esophagus is replaced with the other abnormal tissue and this is the result of long-term acid reflux most of the time. the word low-grade dysplasia refers to starting early abnormal growth of the esophagus lining but it doesn’t directly threaten human life and has a chance of being treated and cured. the highest stage of low-grade dysplasia is called high-grade dysplasia. the inclusion of these different varieties of cancer in the study and testing the performance of the SAVE model toward the WLI is the future direction of this research.

Despite the attainment of favorable results in this research, certain limitations must be acknowledged. The data included in this study was collected from a single medical facility and examined retrospectively. As a result, this may lead to the data being insufficient to capture the heterogeneity present among different hospitals, patients, endoscopic devices, and clinical procedures. The proposed SAVE framework demonstrates effective performance in improving lesion visualization and classification by deep learning, while its therapeutic significance should be approached with care. The suggested strategy will be demonstrated to possess robust generalizability in future studies by verifying it with a multi-center dataset and external validation cohorts. Furthermore, subsequent clinical investigations will be necessary to evaluate the clinical efficacy and application of the SAVE-based computer-aided diagnosis system within conventional endoscopic practice.

For comparing the performance of the SAVE model with the WLI model and analyzing different architecture’s performance in the identification of different types of esophageal cancer, five different ML models were utilized. this comparative technique makes the current study a great benchmark for future studies that may include many more ML models and a larger amount of dataset. the identification of which ML model is more effective in medical image tasking and which model needs further fine-tuning or optimization is done by current analysis. The names of the models that were trained for the given dataset include, YOLOv8, InceptionV3, ConvNeXt-V2, Inception-ResNet-V2, and MobileNetV2. Moreover, this study focuses on sensitivity (recall), precision, and f1-score, future work will incorporate ROC curves analysis to better examine the balance between sensitivity and specificity and to provide AUC-based performance metrics. By addressing this the applicability and reliability of the SAVE model could future improve in the medical sector and lead to accurate identification of esophageal cancer. The results indicate that the SAVE algorithm is a highly efficient, software-driven solution. Its independent processing speed of 120.6 FPS far exceeds the requirements for real-time endoscopy. The integrated pipeline’s performance of 21.4 FPS already approaches the standard video rate (24–30 FPS), confirming that the Spectrum-Aided Vision Enhancer can be effectively integrated into automated screening workflows without introducing significant delays.

The models’ performance varies according to the network configuration and the imaging modalities. While specific models demonstrate superior performance in particular classes or measures, no universal design has been proven to outperform all others across every evaluation criterion. The findings suggest that the enhanced spectral and structural data provided by the SAVE pictures can improve feature representation and classification accuracy across several deep learning models. These results underscore that the efficacy of the specific model is contingent not only upon the network architecture but also on its capacity to leverage the supplementary visual information incorporated during the SAVE augmentation procedure. Despite the high fidelity and improved detection rates achieved by the SAVE framework, this study has certain limitations. First, while we validated the physical characteristics of SAVE against hardware-based NBI using standard benchmarks, we did not perform a direct head-to-head comparison of deep learning classification performance on paired real NBI and SAVE images of the same clinical lesions. This is primarily due to the retrospective nature of our dataset, where paired high-quality WLI and NBI frames for every lesion were not consistently available. Consequently, although SAVE demonstrates significant improvements over WLI, its performance relative to real NBI in an automated CAD system remains to be fully characterized. Future prospective trials involving the simultaneous capture of WLI and NBI data will be essential to establish whether SAVE can fully match or even exceed the diagnostic yields of hardware based NBI in real-world clinical settings.

## Conclusions

In this study, we proposed and validated a novel image enhancement algorithm, the Spectrum-Aided Vision Enhancer (SAVE), designed to overcome the limitations of standard White Light Imaging (WLI) in esophageal cancer detection. By leveraging hyperspectral principles to generate virtual Narrow Band Imaging (NBI) representations, the SAVE mechanism significantly improves the visibility of mucosal vasculature and subtle lesions without the need for specialized hardware. This software-driven approach provides a cost-effective solution for enhancing image quality, which is a critical preprocessing step for automated medical diagnosis. Our comprehensive evaluation using five state-of-the-art deep learning models—YOLOv8, InceptionV3, Inception-ResNet-V2, ConvNeXt-V2, and MobileNetV2—demonstrates that the integration of SAVE consistently outperforms traditional WLI-based training. The models trained on SAVE-enhanced datasets exhibited superior accuracy, sensitivity, and specificity in classifying and detecting Squamous Cell Carcinoma (SCC) and dysplasia. These findings confirm that domain-specific image enhancement can effectively augment the feature extraction capabilities of deep neural networks, leading to more robust and reliable Computer-Aided Diagnosis (CAD) systems. Future work will focus on expanding the dataset to include a wider variety of esophageal pathologies and conducting multi-center clinical trials to further validate the generalizability of the SAVE algorithm. Additionally, we aim to optimize the computational efficiency of the SAVE mechanism for real-time deployment in clinical endoscopic systems. Ultimately, this study highlights the potential of combining advanced image processing techniques with deep learning to assist endoscopists in early cancer detection, potentially reducing missed diagnoses and improving patient outcomes.

## Supplementary Information

Below is the link to the electronic supplementary material.

Supplementary Material 1

## Data Availability

The data presented in this study are available in this article; further considerable requests can be made to the corresponding author (H.-C.W.).
