# Supplementary material for: Enhancing esophageal cancer detection using a deep learning framework and a novel spectrum-aided vision enhancer for virtual narrow band imaging
Source: Sci Rep. 2026 May 20;16:25149. doi: 10.1038/s41598-026-52501-y (PMC13469449; doi:10.1038/s41598-026-52501-y)
Supplement: Supplementary file 1 [file 41598_2026_52501_MOESM1_ESM.docx]

**S1. Different stages and treatment of Esophageal cancer**

Table S1 Summary of recommended treatment options for esophageal cancer at various clinical stages.

| Different stages | The recommended treatment |
| --- | --- |
| Early stages (Tis and T1) | Endoscopic treatment  1 endoscopic mucosal resection (EMR)  2 Endoscopic Submucosal Dissection (ESD) |
| Intermediate stages (T2 and T3) | Esophagectomy surgery  Definitive chemoradiotherapy |
| Advanced stages | Chemoradiotherapy  Targeted therapy e.g. HER2 Inhibitors  Palliative care |

Table S2 shows the image distribution for each class.

| Class name | Number of Patients | Number of the total images per class |
| --- | --- | --- |
| Dysplasia | 50 | 2030 |
| SCC | 65 | 1660 |
| Normal | 70 | 1680 |
| Total | 185 | 5370 |

Table S2 describes the distribution of the total 5370 images into 3 classes for both WLI and SAVE images which are used in the dataset

**S2. Evaluation Indices**

To evaluate the performance of the model various performance metrics such as accuracy, precision, recall (sensitivity or True Positive Rate), and f1 score are calculated based on the confusion matrix.

A. Accuracy: is a measure of total correct prediction done by the model. It is calculated as:

$Accuracy = \frac{TP + TN}{TP + TN + FP + FN}$	(S1)

B. Precision: it indicates how many positive samples are indeed predicted as positive (how many predicted positives are true). The formula is given as:

$Precision = \frac{TP}{TP + FP}$	(S2)

C. Recall: it is also known as (TPR) or Sensitivity which is the ability of the model to capture all the positive cases (completeness of capturing all positive cases). It is given as:

$Recall = \frac{TP}{TP + FN}$	(S3)

D. F1-Score: is the harmonic mean of the precision and recall. The range is between 1 (the best precision and recall) and 0 (the worst precision and recall). And the formula to calculate the F1 score is given as:

$F1-Score = 2\times\frac{Precision \times Recall}{Precision + Recall}$	(S4)

Table S3 Description of parameters TP, TN, FP, FN

| **Parameters** | **Descriptions** |
| --- | --- |
| TP (True Positive) | The number of cancerous tissues (SCC or Dysplasia) that were predicted correctly as SCC or Dysplasia. |
| TN (True Negative) | The number of Normal tissues is predicted as Normal. |
| FP (False Positive) | The number of Normal tissues that were mistakenly identified as SCC or Dysplasia. |
| FN (False Negative) | The number of SCC and dysplasia that were mistakenly predicted as Normal Tissues. |

Table S3 shows the various parameters used in the experiment for the classification of Esophageal cancer to SCC, Dysplasia, and Normal. We have assigned the SCC or Dysplasia as the result which is positive for the test, and Normal as the result which is negative for the test.

**S3. YOLOv8 Result**

YOLOv8 was trained for both the n and x variant each with 300 epochs with the pause value of 100.

Fig S1 shows the architecture diagram of YOLOv8

**YOLOv8n of WLI Images**

**S3.1, Training and validation dataset of WLI**

Fig. S2 The confusion matrix of the 300^th^ epoch of YOLOv8n results for WLI images.

The figure shows the total number of images classified into different classes based on the trained model. The other confusion matrix shows the probability of the model classification into the various classes.

Fig S3 The training accuracy and loss of the model training.

Fig S4 The classification result of the trained model over the validation dataset.

**S3.2, Test dataset OF WLI**

Fig S5 The 300^th^ epoch of YOLOv8n results for WLI images for the test data.

Fig S6 The classification result of the trained model over the test dataset.

**YOLOv8x of WLI Images**

**S3.3, Training and validation dataset of WLI**

Fig S7 The 300^th^ epoch of YOLOv8x results for WLI images for the training and validation dataset.

Fig S8 The training and validation accuracy and loss

Fig S9 The classification result of the trained model over the validation dataset.

**S3.4, Test dataset WLI**

Fig S10 The 300^th^ epoch of YOLOv8x results for WLI images for the test data.

Fig S11 The classification result of the trained model over the test dataset.

**YOLOv8n SAVE images**

**S3.5, Training and validation dataset of SAVE**

Fig S12 The 300^th^ epoch of YOLOv8n results for SAVE images for the training and validation dataset.

Fig S13 The classification result of the trained model over the validation dataset

**S3.6, Test dataset of SAVE**

Fig S14 The 300^th^ epoch YOLOv8n results for SAVE images for the test data.

Fig S15 The classification result of the trained model over the test dataset.

**YOLOv8x SEVE DATA**

Fig S16 The 300^th^ epoch YOLOv8x results for SAVE images for the training and validation dataset.

Fig S17 The training and validation accuracy and loss

Fig S18 The 300^th^ epoch YOLOv8x results for SAVE images for the validation data.

Fig S19 The 300^th^ epoch YOLOv8x results for SAVE images for the test dataset.

Fig S20 The 300^th^ epoch YOLOv8x results for SAVE images for the test data.

**S4. InceptionV3**

**S4.1 WLI Images**

Fig S21 The Accuracy and loss of the trained model

Fig S22 The confusion matrix of the inceptionv3 model for classifying the data into different classes.

Fig S23 The prediction result of the trained model over the test dataset.

**S4.2 SAVE Images**

Fig S24 Training accuracy and loss of the model

Fig S25 confusion which indicates the class-wise classification of the dataset

Fig S26 The prediction result of the trained model over the test dataset.

**S5. Logistic Regression**

**S5.1 WLI Images**

Fig S27 The confusion matrix of the Logistic regression model for the WLI images.

Fig S28 The predicted result of the trained model over the test dataset.

**S5.2 SAVE Images**

Fig S29 The confusion matrix of the Logistic regression model for the SAVE images.

Fig S30 The predicted result of the trained model over test data of SAVE images.

**S6. MobileNetV2**

**S6.1 WLI Images**

Fig S31 The confusion matrix of the MobileNetV2 model for WLI images.

**S6.2 SAVE Images**

Fig S32 The confusion matrix of the MobileNetV2 model for SAVE images.

Fig S33 The prediction result of the trained model over test data of SAVE images.

Fig S34 The training accuracy and validation data accuracy.

Fig S35 The validation and training loss of SAVE images

**S7. VGG16**

**S7.1 WLI Images**

Fig S36 The confusion matrix of VGG16 of WLI images.

Fig S37 The training and validation data accuracy along with loss value.

Fig S38 The prediction result of the trained model over the test data.

**S7.2 SAVE Images**

Fig S39 The confusion matrix of the VGG16 model for SAVE images.

Fig S40 The train and validation data accuracy and loss

Fig S41 The prediction result of the model for the SAVE data.

**S8. Important equations for conversion of WLI to SAVE.**

The primary objective of the SAVE technique is to improve diagnostic capabilities in endoscopy by providing a more detailed spectral analysis than traditional methods. The study emphasizes the calibration of RGB (Red, Green, Blue) images captured by the endoscope to align with reflectance spectra obtained from a spectrometer. This alignment is crucial for accurate spectral information during endoscopic procedures. After converting the WLI images to CIF 1931 XYZ color space and removing all the incorrect color calibration or any other dark currents, the discrete transformation equations which are mentioned in Equation 1 used for converting the 24-color patch picture and 24-color patch reflectance spectrum data to the XYZ color space.

$\left[ \begin{aligned} X \\ Y \\ Z \end{aligned} \right]=[M_{A}]\left[ T \right]\left[ \begin{aligned} f\left( R_{sRGB} \right) \\ f\left( G_{sRGB} \right) \\ f(B_{sRGB}) \end{aligned} \right]\times100 , 0\leq{R_{sRGB} \atop\begin{aligned} G_{sRGB} \\ B_{sRGB} \end{aligned}} \leq1$	(S5)

$\left[ T \right]=\left[ \begin{aligned} 0.4104 0.3576 0.1805 \\ 0.2126 0.7152 0.0722 \\ 0.0193 0.1192 0.9505 \end{aligned} \right]$	(S6)

$f\left( n \right)= \left\{ \begin{aligned} {(\frac{n+0.055}{1.055})}^{2.4}, n>0.04045 \\ \left( \frac{n}{12.92} \right), otherwise \end{aligned} \right.$	(S7)

$\left[ M_{A} \right]=\left[ \begin{aligned} \frac{X_{SW}}{X_{CW}} 0 0 \\ 0 \frac{Y_{SW}}{Y_{CW}} 0 \\ 0 0 \frac{Z_{SW}}{Z_{CW}} \end{aligned} \right]$	(S8)

For the spectrometer component, the reflection spectral data is transformed into the XYZ color space. This allows the spectral data to be adapted to the corresponding color gamut in the XYZ system.

$X=k\int_{400nm}^{700nm} S\left( \lambda\right)R\left( \lambda\right)\bar{x}\left( \lambda\right)d\lambda$	(S9)

$Y=k\int_{400nm}^{700nm} S\left( \lambda\right)R\left( \lambda\right)\bar{y}\left( \lambda\right)d\lambda$	(S10)

$Z=k\int_{400nm}^{700nm} S\left( \lambda\right)R\left( \lambda\right)\bar{z}\left( \lambda\right)d\lambda$	(S11)

$k=100/\int_{400nm}^{700nm} S\left( \lambda\right)\bar{y}\left( \lambda\right)d\lambda$	(S12)

The endoscope’s nonlinear response can be adjusted using a third-order formula, and the variable that represents the modification of the nonlinear response is represented as V_Non-linear._

$V_{Non-linear}=\left[ X^{3} Y^{3} Z^{3} X^{2} Y^{2} Y^{2} X Y Z 1 \right]^{T}$	(S13)

In an imaging device, the dark current remains constant regardless of light levels, so a fixed value is assigned to account for its effect, represented by the variable V_Dark_.

$V_{Dark}=[a]$	(S14)

The variable matrix  V is derived by standardizing the product of V_color_ and V_Non-linear_ including V_Dark_, with the standardization limited to the third order to prevent overcorrection.

$V_{Color}={[XYZ XY XZ YZ X Y Z]}^{T}$	(S15)

$V=\left[ \begin{aligned} X^{3} Y^{3} Z^{3} \\ X^{2}Y X^{2}Z Y^{2}Z \\ XY^{2} XZ^{2} YZ^{2} \\ XYZ X^{2} Y^{2} Y^{2} \\ XY XZ YZ X Y Z a \end{aligned} \right]^{T}$	(S16)

Before applying the CIE DE2000 to calculate color differences, it is necessary to convert XYZCorrect and XYZSpectrum from the XYZ color space to the Lab color space. The conversion follows the equation below.

${L^{*} = 116f\left( \frac{Y}{Y_{n}} \right)-16 \atop\begin{aligned} a^{*} = 500\left[ f(\frac{X}{X_{n}})-f(\frac{Y}{Y_{n}}) \right] \\ b^{*} = 200\left[ f(\frac{Y}{Y_{n}})-f(\frac{Z}{Z_{n}}) \right] \end{aligned}}$	(S17)

$f\left( n \right)= \left\{ \begin{aligned} n^{\frac{1}{3}}, n>0.008856 \\ 7.787n+0.137931, otherwise \end{aligned} \right.$	(S18)

**S9. Hyperparameters and Training Configuration**

To ensure the reproducibility of our experimental results, the detailed hyperparameters and training configurations used for the five deep learning models are summarized in Table S4. All models were implemented using the PyTorch/Keras framework and trained on the hardware described in Section 3.6.

**Table S4. Hyperparameters and training configurations for the deep learning models.**

| Parameter | Configuration / Value |
| --- | --- |
| Transfer Learning | Pre-trained weights from ImageNet were used for all models |
| Input Resolution | 224 × 224 pixels (for classification models); 640 × 640 (for YOLOv8) |
| Batch Size | 32 |
| Optimizer | Adam |
| Initial Learning Rate | 1e-4 |
| Learning Rate Scheduler | Cosine Annealing |
| Training Epochs | 300 epochs for all models |
| Data Augmentation | Horizontal/Vertical Flip, Rotation (±15°), Brightness/Contrast Adjustment |
| Cross-Validation | 5-fold cross-validation |
